# Unlocking the health benefits of melatonin supplementation: A promising preventative and therapeutic strategy

**DOI:** 10.1097/MD.0000000000039657

**Published:** 2024-09-20

**Authors:** Firas S. Azzeh, Waad W. Kamfar, Mazen M. Ghaith, Radi T. Alsafi, Ghalia Shamlan, Mai A. Ghabashi, Wesam F. Farrash, Reema A. Alyamani, Awfa Y. Alazzeh, Sarah O. Alkholy, El-Sayed H. Bakr, Alaa H. Qadhi, Ahmad F. Arbaeen

**Affiliations:** a Department of Clinical Nutrition, Faculty of Applied Medical Sciences, Umm Al-Qura University, Makkah, Saudi Arabia; b Nutrition and Food Services Department, Almana Hospitals, Aziziah, Dammam, Saudi Arabia; c Department of Clinical Laboratory Sciences, Faculty of Applied Medical Sciences, Umm Al-Qura University, AL Abdeyah, Makkah, Saudi Arabia; d Department of Food Science and Nutrition, College of Food and Agriculture Sciences, King Saud University, Riyadh, Saudi Arabia; e Department of Clinical Nutrition, Faculty of Applied Medical Sciences, University of Ha’il, Ha’il, Saudi Arabia.

**Keywords:** anti-inflammatory agent, antioxidant, anti-tumoral activity, COVID-19, melatonin, obesity

## Abstract

Melatonin (MLT) is crucial in controlling human sleep-wake patterns. While it has long been recognized for regulating circadian rhythms, its demonstrated efficacy in managing various diseases has recently gained considerable attention. This review discusses MLT’s potential preventative and therapeutic effects on various diseases. Several studies have focused on examining the molecular mechanisms through which MLT brings about its protective or therapeutic effects on various diseases, including cancer, obesity, coronavirus, and cardiovascular diseases. Numerous preventative and therapeutic applications of MLT have been proposed, resulting from its ability to function as an antioxidant, anti-cancer, anti-inflammatory, and immune-regulating agent. There is a need for further research to determine MLT’s long-term effects on antioxidant defense systems, its preventative and therapeutic benefits, and its molecular basis.

## 1. Introduction

Melatonin (MLT) plays an indispensable role in regulating the circadian rhythm in humans, particularly the sleep cycle.^[1]^ Early studies established that MLT has a sleep-regulating effect as it is mainly produced during the night.^[2]^ Consequently, it is commonly used as a supplement to manage sleep disorders, including insomnia, anxiety, and jet lag.^[3]^

A range of diseases, such as cardiovascular diseases (CVDs), Coronavirus disease (COVID-19), and obesity, have been associated with inadequate secretion of MLT.^[4]^ Studies have demonstrated that MLT possesses robust antioxidant properties, which help protect against oxidative stress. By scavenging free oxygen radicals, MLT can prevent oxidative damage to cells and tissues, which underpins its antioxidant capabilities.^[5–7]^ Additionally, MLT has been linked to boosting the immune system, reducing inflammation, and even potentially helping to prevent certain types of cancer.^[8]^

A considerable amount of research has been dedicated to investigating how MLT can function therapeutically and protectively by regulating human hemostasis through its anti-inflammatory, antioxidant, anti-infective, and anti-tumor properties.^[5–8]^ However, it is necessary to have a comprehensive review that explores the advantages of melatonin, emphasizing its beneficial impact on numerous illnesses. Therefore, this review aims to present the latest discoveries regarding the effectiveness of MLT in promoting health. It not only highlights the potential mechanism through which MLT can contribute to preventing and treating various conditions, including COVID-19, cancer, CVDs, and obesity, but also draws upon the outcomes of both experimental studies and clinical trials. Moreover, this review effectively sheds light on the potential benefits of MLT in mitigating the onset and progression of these illnesses.

## 2. Protective or therapeutic effects of melatonin and its role in various disorders

**Figure 1.**
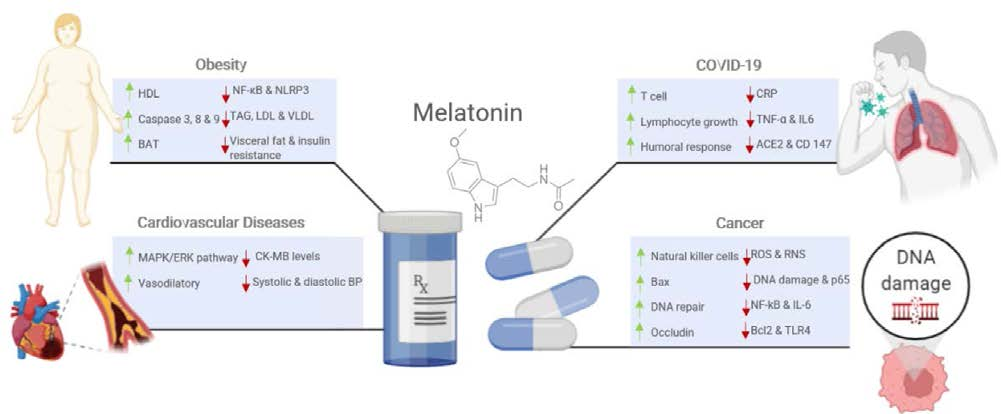
Summary of preventative and therapeutic benefits of melatonin supplement on various pathological conditions. BAT = brown adipose tissue, BP = blood pressure, CD147 = cluster of differentiation, CRP = C-reactive protein, HDL = high-density lipoprotein, LDL = low-density lipoprotein, MAPK/ERK = mitogen-activated protein kinase/extracellular signal-regulated kinase, RNS = reactive nitrogen species, ROS = reactive oxygen species, TAG = triglyceride, TNF-α = tumor necrosis factor alpha, VLDL = very-low-density lipoprotein.

This section provides insight into MLT’s potential benefits regarding the inflammatory and oxidative stress statuses related to various illnesses (Fig. 1). Table 1 also summarizes the therapeutic effects of melatonin and its role in various disorders.

**Table 1:** Therapeutic effects of melatonin and its role in various disorders.

Disease or effect	Main findings	Molecular mechanism	Reference
Anti-inflammatory agent	MLT modulates serum inflammatory parameters	MLT effectively inhibits the activity of NF-κB, a transcription factor that plays a role in regulating pro-inflammatory cytokines	^[9]^
	MLT supplementation could reduce inflammatory biomarkers, such as the inflammation-promoting cytokines TNF-α and IL-6	MLT reduces inflammation in chronic inflammatory diseases by lowering inflammatory mediators, including IL-6, IL-8, cyclooxygenase-2, and iNOS, resulting in a marked improvement in postoperative outcomes	^[10]^
COVID-19	MLT demonstrates a protective mechanism against several pathological consequences of COVID-19, including hemoglobin denaturation, iron accumulation, hypoxia, cardiomyocyte injury, and hypercoagulability	Protective effect by inhibiting the CD147 pathway, and its antioxidant properties in animal models of angiotensin-II-induced cardiac hypertrophy	^[11]^
	A recent case series of 10 patients with COVID-19 pneumonia observed that melatonin supplementation of 36–72 mg/d, administered in 4 divided doses	The findings have shown that MLT has protective effects against SARS-CoV-2 by indirectly inhibiting the SARS-CoV-2 ACE2 receptor coupling, leading to reduced hospital stays, mortality, and mechanical ventilation	^[12]^
	MLT treatment as a prophylactic measure, as a single-drug treatment, or in combination with other drugs to combat SARS-CoV-2 infections. A pharmacological dose of 100–400 mg was recommended as an adjunct to the SARS-CoV-2 treatment	Reducing CRP, TNF-α, and IL6	^[13]^
Cancers			
Anti-tumoral effect	MLT exerts a pro-oxidant action on most cancer cells, stimulating endogenous ROS production with a subsequent increase in DNA damage and cell death	MLT inhibits anti-apoptotic mediators, including NF-κB, preventing Bcl-2 from increasing and reducing inflammatory cytokine production	^[9]^
Breast cancer	MLT has been used in clinical trials as a therapeutic agent in estrogen receptor (ER)-positive breast cancer due to its ability to influence estrogen synthesis and MLT receptors in breast tissue	Reducing ER pathway activation down-regulating the transcription of ER receptors and inhibiting the suppression of aromatase activity	^[14]^
	According to a study on female nurses, urinary aMT6 levels predict the risk of breast cancer for postmenopausal women	A lower concentration of aMT6 has been associated with an increased risk of breast cancer, and this association is not affected by the tumor MLT-1 receptor subtype	^[9]^
Chemotherapy and radiotherapy effect	MLT at a dosage of 20 mg/d may help mitigate the adverse effects of chemotherapy and radiotherapy in cancer patients with solid tumors	Based on the results, MLT used as an adjuvant therapy could enhance complete and partial remission, 1-yr survival, and reduce radiotherapy-related adverse events, such as thrombocytopenia, neurotoxicity, and fatigue	^[15]^
Mammalian gametes and embryos	MLT Reduces peroxide levels and DNA damage consequently improving the viability of germ and embryonic cells	Increasing Bax/Bcl-2 ratios and stimulating apoptosis, MLT also induces these effects by modulating p53, the tumor suppressor oncogene	^[16]^
Ovarian cancer	MLT treatment resulted in a reduced expression of TLRs	Reduced expression of TLRs, specifically TLR-4, NF-κB, IL-6, p65, TLR-7 and TLR-5	^[17]^
Lung cancer	An experiment was conducted to determine the effectiveness of MLT in improving survival rates for patients with metastatic non-small-cell lung carcinoma	Patients received treatment with cisplatin and etoposide, with or without simultaneous administration of MLT. The tumor regression and 5-yr survival rate for patients receiving MLT were significantly higher	^[18]^
Pancreatic cancer	MLT and its metabolite AFMK were found to induce apoptosis in PANC-1 pancreatic cancer cells	Modulating the Bax/Bcl-2 balance, suggesting that they might help improve the therapeutic effects of chemotherapy	^[18]^
CVDs			
CHD	A study of 48 male CHD patients overnight urinary aMT6s levels	Found that low MLT levels at night are associated with an increased risk of ischemic myocardial injury, hypertension, atherosclerosis, HF, and drug-induced myocardial damage	^[19]^
HF	MLT serum levels as a biomarker associated with HF	MLT serum levels negatively correlate with NT-pro-BNP levels	^[9,20]^
MI	Studies have demonstrated that MLT supplementation can reduce the number and size of atheromatous plaques by modulating the ERK pathway	Reversing mitochondrial dysfunction, and decreasing left ventricular remodeling and apoptosis following myocardial infarction	^[21]^
HTN	Supplementation with 2.5 mg/d of MLT for an hour before bedtime for 3 wk	Reduced systolic blood pressure by 6 mm Hg and diastolic blood pressure by 4 mm Hg in 16 men suffering from untreated hypertension	^[22]^
Obesity	MLT exerts an anti-inflammatory effect due to its role as a mitochondrial protector and in preventing insulin resistance	MLT promotes the downregulation of pro-inflammatory plasma cytokines and an upregulation of anti-inflammatory plasma cytokines in animal models of metabolic syndrome	^[23]^
	A recent systematic review and meta-analysis of MLT supplementation of 23 doses of ≤ 8 mg daily	Studies found that 11 reported significant weight loss, BMI, or waist circumference	^[24]^
	MLT-induced apoptosis in preadipocytes by reducing the phosphorylation of ERK and enhancing caspase 3, 8, and 9	Increasing the expression of Bax, a pro-apoptotic protein, MLT has also been demonstrated to reduce the expression of the anti-apoptotic protein Bcl-2	^[9,25]^
	The efficacy of MLT in the treatment of obesity and metabolic syndrome has also been shown in clinical trials of patients suffering from metabolic syndrome, demonstrating that a 5 mg/d	MLT regimen improved dyslipidemia, blood pressure, and antioxidant status	^[9]^
	An experiment was conducted to determine the effects of MLT supplementation in 2 groups of obese subjects who were on a calorie-restricted diet for 30 days, with 10 mg/d MLT supplementation	The results were positive for a significant bodyweight loss in the MLT group; the adiponectin and omentin-1 levels and glutathione peroxidase activities increased, while the malondialdehyde concentrations decreased	^[26]^
	MLT supplementation on a low-calorie diet	Increases the serum levels of omentin-1 in patients	^[27]^

ACE2 = angiotensin-converting enzyme-2, AFMK = N1-actyl-N2-formal-5-methoxykynuramine, BAX = Bcl-2-associated X protein, Bcl-2 = B-cell lymphoma 2, BMI = body mass index, CD147 = cluster of differentiation, CHD = coronary heart disease, COVID-19 = coronavirus disease, COX-2 inhibitors = cyclooxygenase-2, CRP = C-reactive protein, CVDs = cardiovascular diseases, ER = estrogen receptor, ERK = extracellular signal-regulated kinase, HF = heart failure, HTN = hypertension, IL = interleukin, iNOS = nitric oxide synthase, MI = myocardial infarction, MLT = melatonin, NF-κB = nuclear factor-kappa B, NLRP3 = NLR family pyrin domain containing 3, NT-pro-BNP = N-terminal pro-brain natriuretic peptide, SARS-CoV-2 = severe acute respiratory syndrome coronavirus, TLR = toll-like receptor.

### 2.1. Melatonin as an anti-inflammatory agent

Many factors may contribute to inflammation, causing it to occur locally and systematically.^[28]^ MLT has been observed to reduce chronic and acute inflammation by regulating the immune system.^[29]^ According to experimental and clinical research, MLT reduces inflammation in a variety of pathophysiological contexts by influencing pro- and anti-inflammatory cytokines.^[9]^ Numerous investigations have exhibited its capacity to regulate immune system activation by mitigating acute and chronic inflammation.^[17,30]^ Several cytokines are linked to inflammatory illnesses, and the clinical outcomes are partially determined by the ratio of pro- and anti-inflammatory molecules. Thus, MLT may modulate serum inflammatory parameters. MLT effectively inhibits the activity of nuclear factor-kappa B (NF-κB), a transcription factor that plays a role in regulating pro-inflammatory cytokines,^[9]^ modulating its translocation to serve anti-inflammatory roles.^[30]^ Many molecular processes are triggered by NF-κB, and some of these processes could be specific therapeutic targets for the management of inflammation. Furthermore, by reducing inflammatory mediators like interleukin 6 (IL-6), IL-8, cyclooxygenase-2 inhibitors, and inducible nitric oxide synthetase (iNOS), MLT lessens inflammation in chronic inflammatory disorders resulting in a marked improvement in postoperative outcomes.^[10]^ It also inhibits the production of other mediators of inflammation, including chemokines, prostanoids, and C-reactive protein (CRP).^[9]^ A meta-analysis recently concluded that MLT supplementation could reduce inflammatory biomarkers, such as the inflammation-promoting cytokines tumor necrosis factor alpha (TNF-α) and IL-6.^[10]^

### 2.2. Melatonin and COVID-19

On March 11, 2020, the World Health Organization (WHO) declared the Coronavirus pandemic in Wuhan, China, known as the severe acute respiratory syndrome coronavirus 2 (SARS-CoV-2) and responsible for an outbreak of severe respiratory illness named COVID-19. COVID-19 occurs when SARS-CoV-2 binds to angiotensin-converting enzyme 2 (ACE2) receptors in airway epithelial cells, resulting in a pro-inflammatory response and acute respiratory distress syndrome.^[31]^ SARS-CoV-2 can exhibit a range of severity, from individuals who show no symptoms to those who suffer from critical conditions such as acute respiratory distress syndrome and failure of multiple organs.^[13]^

Additionally, the alveoli are damaged by reactive oxygen species (ROS) because of a pro-oxidant response.^[32]^ As stated by Wang et al,^[33]^ there are immunological differences between moderate and severe COVID-19 patients, with the authors demonstrating that the number of CD8 + and CD4 + T cells decrease significantly in severe COVID-19 patients. For patients with moderate COVID-19, the levels of IL-10, IL-6, and TNF-α are within normal limits, whereas they are critically high in severe COVID-19 cases.^[34]^ Macrophages are responsible for producing the cytokines involved in cytokine storm syndrome (CSS), which refers to the cascade of cytokine production resulting from an unregulated host immune response to different stimuli. Triggers include infections, malignancies, and rheumatic diseases;^[35]^ thus, to treat patients with COVID-19, it may be necessary to prevent the CSS.^[34]^

SARS-CoV-2 infection can be prevented by strengthening the immune system to prevent the subsequent lethal COVID-19 episodes.^[13]^ The COVID-19 pandemic has highlighted the crucial role of proper nutrition and dietary practices in mitigating the impact of non-communicable diseases, such as diabetes and obesity, which can exacerbate the severity of infections. Furthermore, maintaining appropriate nutritional habits is essential for managing the inflammatory responses in affected individuals. Neglecting the critical role of nutrition for patients with COVID-19 may adversely influence the effectiveness of nutraceutical products, such as MLT, which are essential for disease susceptibility and maintaining immune health.^[13]^ Using the combination of a healthy lifestyle and nutraceutical products with proven effectiveness, it is possible to prevent some aspects of this infection, thus representing a promising method to combat this condition. A nutraceutical approach to regulating immunity could prove advantageous during the initial stages when a balanced immune response is crucial, as well as in later stages of CSS, where an overly active immune system could have adverse effects.^[13]^

COVID-19 patients treated with MLT demonstrated satisfactory results in terms of possible preventive measures, a predicted decrease in the intensity of their symptoms, and a possible decrease in immunological diseases.^[13]^ MLT has also been demonstrated to modulate innate and adaptive immune reactions, specifically of the inflammasome NLR family pyrin domain containing 3 (NLRP3); the latter pathway is hyperactivated in COVID-19 patients, contributing to CSS.^[13]^ This inflammasome downregulation reduces pulmonary hypertension (HTN), which occurs at critical stages during SARS-COV-2 infection.

It has been observed that MLT interacts with a cluster of differentiation (CD147), a type I transmembrane protein that plays a vital role in viral infection, tumor development, and plasmodium invasion.^[11]^ Current studies suggest that SARS-CoV-2 invades the host cells through the CD147 S protein. However, there is no research conducted yet to determine the effect of MLT on the CD147 S protein in COVID-19 patients. Nonetheless, MLT has been demonstrated to have a protective effect through inhibition of the CD147 signaling pathway, through its antioxidant properties in animal models of angiotensin-II-induced cardiac hypertrophy.^[11]^ This effect has been recognized as a defensive strategy against several pathological outcomes associated with COVID-19, including hemoglobin denaturation, iron accumulation, hypoxia, cardiomyocyte injury, and hypercoagulability.^[11]^ Furthermore, it has been proposed that MLT has protective benefits against the virus due to its indirect inhibition of the SARS-CoV-2 ACE2 receptor coupling; the report concludes that MLT can be used as an adjuvant to boost the efficacy of COVID-19 vaccination.^[36]^

When considering MLT dosage, it should be noted that multiple daily doses may be appropriate to enhance its anti-inflammatory effects when used in conjunction with a virally-induced CSS.^[12]^ A recent case series of 10 patients with COVID-19 pneumonia observed that MLT supplementation of 36 to 72 mg/day administered in 4 divided doses reduced hospital stay, mortality, and mechanical ventilation.^[37]^ Clinical data by Farnoosh et al suggests that MLT exposure had a stronger correlation with the survival of COVID-19 intubated patients.^[38]^ Similarly, a clinical trial conducted by Mehrzadi et al^[39]^ found that patients treated with MLT had faster hospital discharge, quicker return to baseline measures, and significant improvements in clinical and instrumental measures. To combat SARS-CoV-2 infections, Reiter et al,^[34]^ recommended MLT treatment as a prophylactic measure, as a single-drug treatment, or in combination with other medications. A pharmacological dose of 100 to 400 mg was recommended as an adjunct to the SARS-CoV-2 treatment, either immediately following contact with an infected individual or when symptoms begin to manifest. As a result, MLT is effective in reducing CRP, TNF-α, and IL6, with dosages below 25 mg/day. Nonetheless, the appropriate dosage should be determined according to the patient’s age and disease to prevent adverse effects such as drowsiness.^[13]^

### 2.3. Melatonin and anti-tumoral effect

Cancer is a severe life-threatening disease that results in approximately 10 million annual deaths worldwide.^[39]^ It is characterized by dysregulation of the immune system, resulting in a depletion of T cells and natural killer cells, as well as an increment in TNF-α levels.^[40]^ Metastasis occurs due to intricate biological processes and the activation of multiple regulatory enzymes and proteins that are vital for extracellular matrix remodeling and overcoming surrounding tissue barriers to facilitate tumor cell invasion and migration.^[36]^ Targeting the factors contributing to metastasis and invasion can provide anti-cancer properties.^[36]^ The main anti-cancer treatments are chemotherapy, radiotherapy, and surgery.

Research on novel anti-cancer therapies based on herbal extracts and natural compounds has been hindered by the inefficacy and unfavorable side effects of current anti-cancer therapies.^[39]^ Therefore, MLT has received significant focus as a potential treatment alongside radiotherapy and chemotherapy.^[40]^ Considering that cancer initiation is involved in the onset and progression of cancer, the anti-cancer effects of MLT may partly be explained by its antioxidative and anti-inflammatory effects.^[25]^ According to the American Cancer Society, fluctuations in MLT levels in the body can enhance antioxidant functions and stimulate white blood cells, contributing to cancer progression.^[41]^ Furthermore, MLT possesses anti-apoptotic and antioxidant properties capable of annihilating toxic oxygen derivatives, such as ROS.^[17]^ In addition to ROS, MLT also destroys reactive nitrogen species, thus reducing the oxidative and nitrosative damage of macromolecules in all compartments of the cell. MLT also plays an essential role in decimating ROS and reactive nitrogen species levels in mammalian gametes and embryos, which reduces peroxide levels and DNA damage consequently improving the viability of germ and embryonic cells.^[16]^ In addition to increasing Bcl-2-associated X protein (Bax)/B-cell lymphoma 2 (Bcl-2) ratios and stimulating apoptosis, MLT also induces these effects by modulating p53, the tumor suppressor oncogene.^[9]^

MLT regulates apoptosis by varying its actions according to the specific type of cell, whether benign or malignant. Regarding healthy cells, the antioxidant activity of MLT acts by improving the functionality of DNA repair enzymes and reducing cell death in response to radiotherapy and chemotherapy.^[40]^ However, MLT exerts a pro-oxidant action on most cancer cells, stimulating endogenous ROS production with a subsequent increase in DNA damage and cell death. Furthermore, MLT inhibits anti-apoptotic mediators, including NF-κB, preventing Bcl-2 from increasing and reducing inflammatory cytokine production.^[9]^

Several studies have suggested that MLT modulates the secretion of exosomes originating from cancer cells, leading to the suppression of cancer advancement and the modification of lymphocytes and natural killer cells.^[42]^ Moreover, an investigation demonstrated that MLT therapy resulted in a reduced expression of toll-like receptors (TLRs), specifically TLR-4, NF-κB, IL-6, and p65, but also TLR-7 and TLR-5, which play a role in ovarian cancer invasiveness.^[17]^ Recent studies have demonstrated that treatment with MLT in oral cancer CAL27 or SCC25 cell lines resulted in suppression of non-coding micro RNAs, specifically mir-155 and mir-21, which are linked to unfavorable prognosis. Nonetheless, further long-term investigations are essential, along with the examination of additional microRNAs.^[9]^ MLT appears to counteract the invasiveness of non-small cell lung cancer through multiple mechanisms. An experiment was conducted to determine the effectiveness of MLT in improving survival rates for patients with metastatic non-small-cell lung carcinoma. Patients were treated with cisplatin and etoposide, with or without concomitant administration of MLT, and the tumor regression and 5-year survival rate for patients simultaneously receiving MLT were found to be significantly higher.^[18]^ MLT regulates the development of microtubules and microfilaments, leading to the inhibition of the cell cycle via postponing mitosis. Moreover, it upregulates the expression of occludin (an essential protein involved in tight junctions whose downregulation has been linked to the promotion of metastasis). Additionally, MLT subdues epidermal growth factor receptor overexpression, decreases Bcl-2 phosphorylation, and at the same time stimulates Bax. In addition, MLT and its metabolite N1-actyl-N2-formal-5-methoxykynuramine (AFMK) were found to induce apoptosis in PANC-1 pancreatic cancer cells by modulating the Bax/Bcl-2 balance, suggesting that they might help improve the therapeutic effects of chemotherapy.^[18]^ Experimental studies also suggest that MLT may have anti-tumor activity by modulating apoptosis, autophagy, and inflammation, potentially increasing the benefits of chemotherapy by reducing side effects. Preliminary clinical studies have reported that MLT may have beneficial effects on patients with gastrointestinal tumors, either alone or in combination with other therapeutic agents.^[5]^ Furthermore, new evidence suggests that the pharmacodynamics of MLT’s anti-proliferative activity depends on the time of drug administration, with the highest efficacy being observed when administered late in the light phase.^[15]^ This anti-tumor effect may be attributed to the downregulation of specific hormones that contribute to the growth of tumor cells, including gonadal estrogens, MLT-mediated immune enhancement, the antioxidant potential of indole derivatives, modulation of the cell cycle, induction of apoptosis, and the direct inhibition of cell proliferation by telomerase activity. MLT has been utilized in clinical trials as a treatment for estrogen receptor (ER)-positive breast cancer, owing to its capacity to modulate estrogen formation and MLT receptors within breast tissue. This intervention has been shown to diminish the activation of the ER pathway and decrease the transcription of ER receptors. Interestingly, aromatase activity is regulated by MLT in breast cancer cells, with estrogen biosynthesis being inhibited by the suppression of aromatase activity.^[43]^ In this regard, working night shifts is associated with an elevated risk of breast cancer due to the suppression of MLT formation caused by exposure to light at night. In breast cancer patients undergoing chemotherapy, the circulating levels of MLT and AFMK were lower compared to healthy women, independent of sleep patterns.^[14]^ According to an investigation concerning female nurses, it was observed that concentration of urinary aMT6 can serve as a predictive indicator for the risk of postmenopausal breast cancer. Specifically, a lower concentration of aMT6 has been linked to a higher risk of breast cancer. Importantly, this correlation is not affected by the tumor MLT-1 receptor subtype.^[9]^ Clinical trials with a larger sample size are necessary to confirm the findings of these studies.

Immunomodulating neurohormones normally regulate the release of hormones and cytokines by the endocrine system. Accordingly, as an immunomodulatory agent, MLT may be considered a palliative treatment for cachexia.^[15]^ The effects of MLT on tumors can be classified as either cytostatic or cytotoxic. Cytotoxicity occurs exclusively at high MLT concentrations, and the type of cells and tumor determine the level of effectiveness. In this respect, MLT treatment levels may differ based on the type of cancer cell. Breast cancer cells, for example, are sensitive to low (nanomolecular) MLT concentrations, stopping their proliferation. Other cancer cells do not respond to low MLT levels, whilst high MLT levels can inhibit their proliferation, as observed in colon cancer cells and human prostate cancer cells. The absence of cytotoxicity is observed in all nontumor cells, even at high MLT levels. Due to this property, MLT can be used as an anti-tumor agent without causing damage to healthy cells.^[44]^ According to the systematic review and meta-analysis of 8 RCTs by Wang et al,^[33]^ MLT may reduce the adverse effects of chemotherapy and radiotherapy among cancer patients with solid tumors. MLT was administered at 20 mg/day in all of these studies. Based on the results, MLT as an adjuvant therapy could improve complete and partial remission, 1-year survival, and radiotherapy-related adverse events, including thrombocytopenia, neurotoxicity, and fatigue.^[15]^

### 2.4. Melatonin and cardiovascular diseases

CVDs are the leading cause of mortality worldwide; therefore, preventative measures are necessary to avoid the creation and development of these disorders.^[45]^ Heart failure (HF) is primarily caused by ischemic heart disease, which is caused by an imbalance between cardiac blood supply and myocardial oxygen and nutritional requirements, leading to myocardial ischemia.^[46]^ Additionally, this is related to the insufficient removal of metabolic end-products, resulting in cardiomyocyte loss through necrosis, necroptosis, apoptosis, or autophagy, followed by reparative fibrotic healing, ventricular remodeling, and eventually, HF.^[47]^

MLT is associated with the regulation of several cardiovascular parameters, including blood pressure, and is considered a potential antihypertensive agent.^[19]^ Evidence has grown in the last 2 decades to indicate that MLT deficiency contributes to various CVDs.^[48]^ Researchers have demonstrated that low MLT levels at night are related to a higher likelihood of experiencing atherosclerosis, ischemic myocardial injury, HTN, HF, and medication-induced damage to the heart.^[19]^ A study of 48 male coronary heart disease (CHD) patients found that overnight urinary aMT6s levels were significantly lower than in the control group.^[19]^ In another study, nighttime serum MLT levels in CHD patients were approximately 5 times lower than in healthy controls.^[48]^ Similarly, MLT secretion was lower in 16 patients with coronary artery disease at 2 am, 4 am, and 8 am compared to controls.^[19]^ Comparing levels of urinary aMT6s between patients with stable and unstable angina and coronary disease, nocturnal urinary aMT6s levels in patients with unstable angina were significantly lower than in both healthy controls and patients with stable angina.^[48]^

Ghaeli et al^[49]^ suggested that individuals who experience ST-segment elevation myocardial infarction (MI) usually receive primary percutaneous coronary intervention. The use of MLT as a standard treatment approach notably decreased the level of creatine kinase-MB compared to a group that received only traditional treatment.^[48]^ Investigations propose that MLT levels in the blood may serve as an important indicator of HF. Researchers found that treatments with MLT and ACE2 inhibitors could relieve the pathological changes observed in rats exposed to prolonged light exposure, including HTN, enlargement of the left ventricle, and hardening of the left ventricle and aorta, along with increased oxidative stress.^[45]^ Additionally, both the release and circulating levels of MLT are diminished in patients with both acute and chronic HF.^[46]^ Importantly, blood MLT levels show an inverse relationship with N-terminal pro-brain natriuretic peptide levels, which is a marker associated with HF.^[9,20]^

Exogenous MLT administration has profound protective effects against several cardiac diseases, including ischemia-reperfusion injury, diabetic cardiomyopathy, HF, aluminum phosphide-induced cardiotoxicity, 2,3,7,8-tetrachlorodibenzo-p-dioxin-induced cardiac injury, elevated heart rate, and postural tachycardia syndrome.^[45]^ Through modifying the extracellular signal-regulated kinase (ERK) pathway, reversing mitochondrial dysfunction, and decreasing left ventricular remodeling and apoptosis following MI, a study has shown that MLT supplementation can decrease the quantity and size of atheromatous plaques.^[21]^ According to clinical studies, MLT attenuates complications of MI, as well as the effects of myocardial damage caused by ischemia and ventricle hypertrophy. It also serves as a complementary treatment alongside non-surgical and surgical interventions for CVDs.^[50]^

A double-blind, placebo-controlled study concluded that 2.5 mg/day of MLT for 3 weeks significantly reduced both systolic and diastolic blood pressure in hypertensive patients.^[51]^ In a related study, MLT could also reduce blood pressure, circulating catecholamines, and vascular reactivity in healthy volunteers.^[51]^ According to a systematic review and meta-analysis of 8 randomized controlled trials, MLT administration significantly lowers systolic and diastolic blood pressure in patients with metabolic disorders.^[52]^ In a study by Lee et al,^[22]^ supplementation with 2.5 mg/day an hour before bedtime for 3 weeks reduced systolic blood pressure by 6 mm Hg and diastolic blood pressure by 4 mm Hg in 16 men suffering from untreated HTN. Similarly, Koziróg et al^[53]^ found that supplemental MLT at a dose of 5 mg/day taken 2 hours before bedtime for 2 months reduced systolic blood pressure by nearly 13 mm Hg and diastolic blood pressure by 6.5 mm Hg in metabolic syndrome patients who were unresponsive to lifestyle interventions. In addition, the authors concluded that 2 to 10 mg/day of MLT supplementation during intervention periods ranging from 4 to 12 weeks reduced systolic blood pressure by 0.87 mm Hg and diastolic blood pressure by 0.85 mm Hg among patients with metabolic disorders.^[53]^ Bazyar et al,^[54]^ found that supplemental MLT is efficient in managing blood pressure in diabetic patients.

### 2.5. Melatonin and obesity

Obesity is a global problem and poses a significant public health challenge in the 21st century.^[55]^ In industrialized nations, obesity is becoming a serious health issue due to unhealthy lifestyle habits, such as sedentary behaviors and fat-rich diets.^[9]^ It results from excess calories in the diet stored in the white adipose tissue (WAT) in the form of triglycerides.^[56]^

Obesity is known to be characterized by excessive oxidative stress and chronic inflammation. It has been linked to chronic inflammation mediated by cytokines expressed in adipose tissue, such as IL-6 and TNF-α.^[9,57]^ As a result of increased fat mass, one of 2 processes is required: adipocyte hypertrophy or adipocyte hyperplasia through *de novo* differentiation from progenitors. Adipocyte hypertrophy is known to cause morbid obesity;^[56,23]^ An essential characteristic of this condition is the enlargement of fat cells in fat depots, a high level of M1 macrophage infiltration, limited vessel development, and massive fibrosis.^[58]^ Due to these facts, pathological expansions are associated with chronic inflammation and dysfunction of the WAT. The difficulties associated with WAT dysfunction are considered one of the leading causes of obesity-related medical complications. WAT is one of the first to develop inflammatory responses, which are initiated by classical inflammatory pathways and result in the infiltration of macrophages, neutrophils, and lymphocytes, leading to the generation of multiple pro-inflammatory mediators, which eventually contribute to systemic insulin resistance.^[59]^

Oxidative stress stimulates the conversion of preadipocytes into adipocytes.^[24]^ This process is specifically triggered by hydrogen peroxide, which positively regulates the transcriptional activators essential for adipocyte differentiation, such as CCAAT/enhancer binding protein-beta and peroxisome proliferator-activated receptor-gamma.^[9]^ It has been demonstrated that disruption of adipokine secretion is a significant factor in the pathophysiology of metabolic disorders associated with obesity,^[23]^ potentially linked to varying levels of adipokine excretion in visceral and subcutaneous WAT deposits.^[9]^ Energy production and expenditure are regulated by adipokines such as adiponectin, and resistin. Adiponectin is a hormone that exerts anti-inflammatory effects and is associated with various physiological conditions related to lipid and glucose metabolism and appetite.^[23]^ Subcutaneous WAT produces greater adiponectin than visceral WAT. Bodyweight and adiponectin were correlated negatively. In individuals with obesity, the concentration of adiponectin diminishes, whereas it tends to rise during weight reduction.^[23]^ Omentin-1 is another insulin-sensitizing and anti-inflammatory adipokine produced by visceral WAT and improves insulin-stimulated glucose uptake by adipocytes. There is evidence that the levels of omentin-1 in the body decrease in obese individuals and are negatively linked to metabolic syndrome.^[9]^

Several studies have demonstrated a link between MLT deficiency and obesity, or the potential role of MLT in preventing obesity and its complications.^[58,60,61]^ Many therapeutic strategies have been developed to improve the condition caused by this tissue dysfunction. MLT could have a significant positive impact through its antioxidant properties and regulation of metabolism.^[9]^ In addition to epigenetically modulating nuclear factor-erythroid factor 2-related factor 2, MLT has anti-inflammatory effects by inhibiting NF-κB and NLR family pyrin domain containing 3.^[27,62]^ The pineal hormone has been proven to have beneficial effects in the treatment of obesity-related characteristics, including a decrease in the adipocyte secretion of TNF-α and IL-6, an increase in high-density lipoprotein cholesterol, a reduction in visceral fat, and a reduction of plasma levels of triglyceride, low-density lipoproteins, very-low-density lipoprotein cholesterol.^[62]^ Several studies have demonstrated that MLT, responsible for the synchronization of many physiological effects, may be beneficial in managing obesity and its complications; it has also been reported to significantly affect energy metabolism,^[55,58]^ while insulin has been demonstrated to significantly impact glucose and lipid metabolism.^[24]^ Most of these studies involve WAT in rodents.^[55]^ Furthermore, there are chronobiological aspects of MLT and its relationship with cytokines produced by WAT, such as leptin and adiponectin.^[55,23]^ It has also been documented that MLT exerts an anti-inflammatory effect due to its role as a mitochondrial protector and in preventing insulin resistance. It promotes the downregulation of pro-inflammatory plasma cytokines and an upregulation of anti-inflammatory plasma cytokines in animal models of metabolic syndrome.^[23]^ Thus, there is substantial evidence to support MLT in treating and preventing the complications of obesity.

In a recent study, MLT-induced apoptosis in preadipocytes by reducing the phosphorylation of ERK and enhancing caspase 3, 8, and 9.^[25]^ Instead of upregulating Bax, a protein that promotes apoptosis, MLT has also been shown to downregulate the anti-apoptotic protein Bcl-2.^[9]^ MLT has recently been suggested as a weight loss supplement in humans because it can boost brown adipose tissue (BAT) growth and metabolic activity. Additionally, MLT may be an excellent strategy for decreasing obesity by stimulating BAT, a metabolically active organ that converts excess energy into heat.^[26]^

The efficacy of MLT in the treatment of obesity and metabolic syndrome has also been shown in clinical trials of patients suffering from metabolic syndrome, demonstrating that a 5 mg/day MLT regimen improved dyslipidemia, blood pressure, and antioxidant status.^[9]^ An investigation was carried out to evaluate the impacts of MLT supplementation in 2 groups of obese subjects who were on a calorie-restricted diet for a month, with 10 mg/day MLT supplementation in the MLT group and placebo supplementation in the control group. The results were positive for a significant bodyweight loss in the MLT group; the adiponectin and omentin-1 levels and glutathione peroxidase activities increased, while the malondialdehyde concentrations decreased.^[26]^ The findings of this study indicated that MLT reduces oxidative stress and may suggest a valuable therapeutic strategy for obese patients, as excess oxidative stress is associated with increased insulin resistance and abdominal fat accumulation.^[23]^ MLT supplementation increases the serum levels of omentin-1 in patients adhering to a low-calorie dietary regimen.^[27]^ In healthy young women, higher levels of nocturnal MLT secretion were also linked to reduced occurrence of insulin resistance.^[62]^

Human studies have examined the link between MLT and BAT activity.^[26]^ Three mg MLT administered daily for 3 months increased BAT volume in 4 patients diagnosed with MLT deficiency due to radiation therapy or surgical removal of the pineal gland.^[26]^ In addition, a recent systematic review and meta-analysis of 23 studies found that 11 reported significant weight loss, BMI, or waist circumference results from MLT supplementation compared to placebos, and the results were better in studies with doses of ≤ 8 mg daily.^[24]^

## 3. Conclusion

This review discusses the potential preventive and therapeutic benefits of MLT for different diseases, including cancer, obesity, infection, and cardiovascular diseases. MLT has been found to have various health advantages, including acting as an antioxidant, a substance with anti-cancer effects, an anti-inflammatory agent, and regulator of immune response. Consequently, MLT has been applied in both prevention and treatment. More clinical and experimental research is needed to examine the effects of MLT on chronic diseases, its therapeutic and preventive benefits, and its molecular mechanisms. It is also essential to study the effect of MLT on the spike protein in COVID-19 patients and to determine the appropriate dosage based on the patient’s age and clinical condition to prevent adverse effects such as drowsiness. Long-term studies and consideration of other mi-RNAs are still necessary. The appropriate dosage should be determined according to the patient’s age and clinical condition to prevent adverse effects such as drowsiness, but it is still necessary to conduct long-term studies and consider other mi-RNAs.

## Author contributions

**Conceptualization:** Firas S. Azzeh, Mazen M. Ghaith.

**Data curation:** Firas S. Azzeh, Waad W. Kamfar.

**Formal analysis:** Firas S. Azzeh, Waad W. Kamfar, El-Sayed H. Bakr, Mai A. Ghabashi, Sarah O. Alkholy.

**Investigation:** Firas S. Azzeh, Waad W. Kamfar, Mai A. Ghabashi, Wesam F. Farrash, Sarah O. Alkholy.

**Methodology:** Firas S. Azzeh, Waad W. Kamfar, Mai A. Ghabashi, Wesam F. Farrash, Sarah O. Alkholy, Alaa H. Qadhi.

**Project administration:** Firas S. Azzeh, Waad W. Kamfar, Awfa Y. Alazzeh.

**Resources:** Firas S. Azzeh, Waad W. Kamfar, Ghalia Shamlan, El-Sayed H. Bakr, Wesam F. Farrash, Reema A. Alyamani, Sarah O. Alkholy, Alaa H. Qadhi.

**Software:** Firas S. Azzeh, Waad W. Kamfar, Mazen M. Ghaith, Wesam F. Farrash, Reema A. Alyamani, Awfa Y. Alazzeh, Alaa H. Qadhi.

**Supervision:** Firas S. Azzeh, Mazen M. Ghaith, Radi T. Alsafi, Ghalia Shamlan, Reema A. Alyamani, Awfa Y. Alazzeh, Alaa H. Qadhi.

**Validation:** Firas S. Azzeh, Mazen M. Ghaith, Radi T. Alsafi, Ghalia Shamlan, El-Sayed H. Bakr.

**Visualization:** Firas S. Azzeh, Radi T. Alsafi, Ghalia Shamlan, El-Sayed H. Bakr.

**Writing – original draft:** Firas S. Azzeh, Waad W. Kamfar, Mazen M. Ghaith, Mai A. Ghabashi, Ahmad F. Arbaeen.

**Writing – review & editing:** Firas S. Azzeh, Waad W. Kamfar, Mazen M. Ghaith, Radi T. Alsafi, Ghalia Shamlan, El-Sayed H. Bakr, Mai A. Ghabashi, Wesam F. Farrash, Reema A. Alyamani, Awfa Y. Alazzeh, Sarah O. Alkholy, Alaa H. Qadhi, Ahmad F. Arbaeen.

## References

[1] Pereira N Naufel MF Ribeiro EB Tufik S Hachul H. Influence of dietary sources of melatonin on sleep quality: a review. J Food Sci. 2020;85:5–13. 31856339 10.1111/1750-3841.14952

[2] Qari SH Hassan MU Chattha MU. Melatonin induced cold tolerance in plants: physiological and molecular responses. Front Plant Sci. 2022;13:843071. 35371159 10.3389/fpls.2022.843071 PMC8967244

[3] Alshehri FS Alghamdi BS Hakami AY Alshehri AA Althobaiti YS. Melatonin attenuates morphine-induced conditioned place preference in Wistar rats. Brain Behav. 2021;11:e2397. 34710287 10.1002/brb3.2397 PMC8671767

[4] Minich DM Henning M Darley C Fahoum M Schuler CB Frame J. Is Melatonin the “Next Vitamin D?”: a review of emerging science, clinical uses, safety, and dietary supplements. Nutrients. 2022;14:3934. 36986237 10.3390/nu15061507 PMC10053200

[5] Talib WH Alsayed AR Abuawad A Daoud S Mahmod AI. Melatonin in cancer treatment: current knowledge and future opportunities. Molecules. 2021;26:2506. 33923028 10.3390/molecules26092506 PMC8123278

[6] Vlachou M Siamidi A Dedeloudi A Konstantinidou S Papanastasiou I. Pineal hormone melatonin as an adjuvant treatment for COVID-19. Int J Mol Med. 2021;47:1. 10.3892/ijmm.2021.4880 PMC7891824 33576451

[7] Rebollo-Hernanz M Aguilera Y Herrera T. Bioavailability of melatonin from lentil sprouts and its role in the plasmatic antioxidant status in rats. Foods. 2020;9:330. 32178261 10.3390/foods9030330 PMC7143261

[8] Posadzki PP Bajpai R Kyaw BM. Melatonin and health: an umbrella review of health outcomes and biological mechanisms of action. BMC Med. 2018;16:1–18. 10.1186/s12916-017-1000-8 PMC5798185 29397794

[9] Ferlazzo N Andolina G Cannata A. Is melatonin the cornucopia of the 21st century? Antioxidants. 2020;9:1088. 33167396 10.3390/antiox9111088 PMC7694322

[10] Zarezadeh M Khorshidi M Emami M. Melatonin supplementation and pro-inflammatory mediators: a systematic review and meta-analysis of clinical trials. Eur J Nutr. 2020;59:1803–13. 31679041 10.1007/s00394-019-02123-0

[11] Sehirli AO Sayiner S Serakinci N. Role of melatonin in the treatment of COVID-19; as an adjuvant through cluster differentiation 147 (CD147). Mol Biol Rep. 2020;47:8229–33. 32920757 10.1007/s11033-020-05830-8 PMC7486968

[12] Cardinali DP Brown GM Pandi-Perumal SR. Can melatonin be a potential “silver bullet” in treating COVID-19 patients? Diseases. 2020;8:44. 33256258 10.3390/diseases8040044 PMC7709121

[13] Corrao S Mallaci Bocchio R Lo Monaco M. Does evidence exist to blunt inflammatory response by nutraceutical supplementation during COVID-19 pandemic? An overview of systematic reviews of vitamin D, vitamin C, melatonin, and zinc. Nutrients. 2021;13:1261. 33921297 10.3390/nu13041261 PMC8069903

[14] Lee HE Lee J Jang TW Kim IA Park J Song J. The relationship between night work and breast cancer. Ann Occup Environ Med. 2018;30:11. 29445504 10.1186/s40557-018-0221-4 PMC5801774

[15] Iravani S Eslami P Dooghaie Moghadam A. The role of melatonin in colorectal cancer. J Gastrointest Cancer. 2020;51:748–53. 31792737 10.1007/s12029-019-00336-4

[16] Farhood B Goradel NH Mortezaee K Khanlarkhani N Najafi M Sahebkar A. Melatonin and cancer: from the promotion of genomic stability to use in cancer treatment. J Cell Physiol. 2019;234:5613–27. 30238978 10.1002/jcp.27391

[17] Tamtaji OR Reiter RJ Alipoor R Dadgostar E Kouchaki E Asemi Z. Melatonin and Parkinson disease: current status and future perspectives for molecular mechanisms. Cell Mol Neurobiol. 2020;40:15–23. 31388798 10.1007/s10571-019-00720-5 PMC11448849

[18] Gurunathan S Qasim M Kang MH Kim JH. Role and therapeutic potential of melatonin in various type of cancers. Onco Targets Ther. 2021;14:2019–52. 33776451 10.2147/OTT.S298512 PMC7987311

[19] Imenshahidi M Karimi G Hosseinzadeh H. Effects of melatonin on cardiovascular risk factors and metabolic syndrome: a comprehensive review. Naunyn Schmiedebergs Arch Pharmacol. 2020;393:521–36. 32002576 10.1007/s00210-020-01822-4

[20] Cao Z Jia Y Zhu B. BNP and NT-proBNP as diagnostic biomarkers for cardiac dysfunction in both clinical and forensic medicine. Int J Mol Sci . 2019;20:1820. 31013779 10.3390/ijms20081820 PMC6515513

[21] Fu Z Jiao Y Wang J. Cardioprotective role of melatonin in acute myocardial infarction. Front Physiol. 2020;11:366. 32411013 10.3389/fphys.2020.00366 PMC7201093

[22] Lee EK Poon P Yu CP Lee VW Chung VC Wong SY. Controlled-release oral melatonin supplementation for hypertension and nocturnal hypertension: a systematic review and meta-analysis. J Clin Hypertens (Greenwich). 2022;24:529–35. 35388609 10.1111/jch.14482 PMC9106086

[23] Karolczak K Watala C. The mystery behind the pineal gland: melatonin affects the metabolism of cholesterol. Oxid Med Cell Longev. 2019;2019:4531865. 31360294 10.1155/2019/4531865 PMC6652030

[24] Guan Q Wang Z Cao J Dong Y Chen Y. Mechanisms of melatonin in obesity: a review. Int J Mol Sci . 2021;23:218. 35008644 10.3390/ijms23010218 PMC8745381

[25] Samec M Liskova A Koklesova L. Metabolic anti-cancer effects of melatonin: Clinically relevant prospects. Cancers. 2021;13:3018. 34208645 10.3390/cancers13123018 PMC8234897

[26] Karamitri A Jockers R. Melatonin in type 2 diabetes mellitus and obesity. Nat Rev Endocrinol. 2019;15:105–25. 30531911 10.1038/s41574-018-0130-1

[27] Genario R Cipolla-Neto J Bueno AA Santos HO. Melatonin supplementation in the management of obesity and obesity-associated disorders: a review of physiological mechanisms and clinical applications. Pharmacol Res. 2021;163:105254. 33080320 10.1016/j.phrs.2020.105254

[28] Meng X Li Y Li S. Dietary sources and bioactivities of melatonin. Nutrients. 2017;9:367. 28387721 10.3390/nu9040367 PMC5409706

[29] Salehi B Sharopov F Fokou PVT. Melatonin in medicinal and food plants: occurrence, bioavailability, and health potential for humans. Cells. 2019;8:681. 31284489 10.3390/cells8070681 PMC6678868

[30] Tarocco A Caroccia N Morciano G. Melatonin as a master regulator of cell death and inflammation: molecular mechanisms and clinical implications for newborn care. Cell Death Dis. 2019;10:1–12. 10.1038/s41419-019-1556-7 PMC6453953 30962427

[31] Romero A Ramos E López-Muñoz F Gil-Martín E Escames G Reiter RJ. Coronavirus disease 2019 (COVID-19) and its neuroinvasive capacity: is it time for melatonin? Cell Mol Neurobiol. 2022;42:489–500. 32772307 10.1007/s10571-020-00938-8 PMC7415199

[32] Cross KM Landis DM Sehgal L Payne JD. Melatonin for the early treatment of COVID-19: a narrative review of current evidence and possible efficacy. Endocr Pract. 2021;27:850–5. 34119679 10.1016/j.eprac.2021.06.001 PMC8190272

[33] Wang XC Wu GL Cai YF Zhang SJ. The safety and efficacy of melatonin in the treatment of COVID-19: a systematic review and meta-analysis. Medicine (Baltim). 2022;101:e30874. 10.1097/MD.0000000000030874 PMC9524532 36181086

[34] Reiter RJ Abreu-Gonzalez P Marik PE Dominguez-Rodriguez A. Therapeutic algorithm for use of melatonin in patients with COVID-19. Front Med. 2020;7:226. 10.3389/fmed.2020.00226 PMC7242729 32574327

[35] Tang Y Liu J Zhang D Xu Z Ji J Wen C. Cytokine storm in COVID-19: the current evidence and treatment strategies. Front Immunol. 2020;11:1708. 32754163 10.3389/fimmu.2020.01708 PMC7365923

[36] Maroufi NF Ashouri N Mortezania Z. The potential therapeutic effects of melatonin on breast cancer: an invasion and metastasis inhibitor. Pathol Res Pract. 2020;216:153226. 32987338 10.1016/j.prp.2020.153226

[37] Camp OG Bai D Gonullu DC Nayak N Abu-Soud HM. Melatonin interferes with COVID-19 at several distinct ROS-related steps. J Inorg Biochem. 2021;223:111546. 34304092 10.1016/j.jinorgbio.2021.111546 PMC8285369

[38] Farnoosh G Akbariqomi M Badri T. Efficacy of a low dose of melatonin as an adjunctive therapy in hospitalized patients with covid-19: a randomized, double-blind clinical trial. Arch Med Res. 2022;53:79–85. 34229896 10.1016/j.arcmed.2021.06.006 PMC8220995

[39] Mehrzadi S Pourhanifeh MH Mirzaei A Moradian F Hosseinzadeh A. An updated review of mechanistic potentials of melatonin against cancer: pivotal roles in angiogenesis, apoptosis, autophagy, endoplasmic reticulum stress and oxidative stress. Cancer Cell Int. 2021;21:188. 33789681 10.1186/s12935-021-01892-1 PMC8011077

[40] Wang L Wang C Choi WS. Use of melatonin in cancer treatment: where are we? Int J Mol Sci . 2022;23:3779. 35409137 10.3390/ijms23073779 PMC8998229

[41] Bhattacharya S Patel KK Dehari D Agrawal AK Singh S. Melatonin and its ubiquitous anticancer effects. Mol Cell Biochem. 2019;462:133–55. 31451998 10.1007/s11010-019-03617-5

[42] Moloudizargari M Moradkhani F Hekmatirad S Fallah M Asghari MH Reiter RJ. Therapeutic targets of cancer drugs: modulation by melatonin. Life Sci. 2021;267:118934. 33385405 10.1016/j.lfs.2020.118934

[43] Jin Y Choi YJ Heo K Park SJ. Melatonin as an oncostatic molecule based on its anti-aromatase role in breast cancer. Int J Mol Sci . 2021;22:438. 33406787 10.3390/ijms22010438 PMC7795758

[44] Kubatka P Zubor P Busselberg D. Melatonin and breast cancer: evidences from preclinical and human studies. Crit Rev Oncol Hematol. 2018;122:133–43. 29458781 10.1016/j.critrevonc.2017.12.018

[45] Odah MM Alfakieh HOH Almathami AA Almuashi HIM Awad MAH Ewis AA. Public awareness of coronary artery disease and its risk factors among Al-Qunfudah Governorate population. J Umm Al-Qura Univ Med Sci. 2022;8:34–8.

[46] Nduhirabandi F Maarman GJ. Melatonin in heart failure: a Promising therapeutic strategy? Molecules. 2018;23:1819. 30037127 10.3390/molecules23071819 PMC6099639

[47] Radwan RA Alwafi HH Alhindi YZ. Patterns of caffeine consumption in Western Province of Saudi Arabia. Pharmacognosy Research. 2022;14:269–75.

[48] Nishi T Saeki K Miyata K. Effects of cataract surgery on melatonin secretion in adults 60 years and older: a randomized clinical trial. JAMA Ophthalmol. 2020;138:405–11. 32134436 10.1001/jamaophthalmol.2020.0206 PMC12530708

[49] Ghaeli P Vejdani S Ariamanesh A Hajhossein Talasaz A. Effect of melatonin on cardiac injury after primary percutaneous coronary intervention: a randomized controlled trial. Iran J Pharm Res. 2015;14:851–5. 26330873 PMC4518113

[50] Lu L Ma J Sun M. Melatonin ameliorates MI-induced cardiac remodeling and apoptosis through a JNK/p53-dependent mechanism in diabetes mellitus. Oxid Med Cell Longev. 2020;1535201:1–14. 10.1155/2020/1535201 PMC7199622 32411318

[51] Pandi-Perumal SR BaHammam AS Ojike NI. Melatonin and human cardiovascular disease. J Cardiovasc Pharmacol Ther. 2017;22:122–32. 27450357 10.1177/1074248416660622

[52] Akbari M Ostadmohammadi V Mirhosseini N. The effects of melatonin supplementation on blood pressure in patients with metabolic disorders: a systematic review and meta-analysis of randomized controlled trials. J Hum Hypertens. 2020;34:413. 31645639 10.1038/s41371-019-0278-8

[53] Koziróg M Poliwczak AR Duchnowicz P Koter-Michalak M Sikora J Broncel M. Melatonin treatment improves blood pressure, lipid profile, and parameters of oxidative stress in patients with metabolic syndrome. J Pineal Res. 2011;50:261–6. 21138476 10.1111/j.1600-079X.2010.00835.x

[54] Bazyar H Zare Javid A Bavi Behbahani H Moradi F Moradi Poode B Amiri P. Consumption of melatonin supplement improves cardiovascular disease risk factors and anthropometric indices in type 2 diabetes mellitus patients: a double-blind, randomized, placebo-controlled trial. Trials. 2021;22:231. 33766084 10.1186/s13063-021-05174-z PMC7995760

[55] Zia T Naveel T Arshad S. An insight into obesity and overweight frequency secondary to unhealthy dietary intake among housewives in Pakistan. Pakistan J Med Health Sci. 2022;16:877–8.

[56] Islam MA Al-karasneh AF Hussain AB. Assessment of beverage consumption by young adults in Saudi Arabia. Saudi Pharm J. 2020;28:1635–47. 33424256 10.1016/j.jsps.2020.10.010 PMC7783230

[57] Alafari H Alshaya DS Alnaam Y. (2022). Relationship between obesity and immunological parameters among students at the PSAU University-Alkharj, KSA. Int J Health Sci. 2022;6:1142–50.

[58] Muscogiuri G Barrea L Annunziata G. Obesity and sleep disturbance: the chicken or the egg? Crit Rev Food Sci Nutr. 2019;59:2158–65. 30335476 10.1080/10408398.2018.1506979

[59] de Farias TDSM Cruz MM de Sa RCC. Melatonin supplementation decreases hypertrophic obesity and inflammation induced by high-fat diet in mice. Front Endocrinol. 2019;10:750. 10.3389/fendo.2019.00750 PMC6848267 31749764

[60] Szewczyk-Golec K Rajewski P Gackowski M. Melatonin supplementation lowers oxidative stress and regulates adipokines in obese patients on a calorie-restricted diet. Oxid Med Cell Longev. 2017;8494107:1–10. 10.1155/2017/8494107 PMC5632922 29142618

[61] Cipolla-Neto J Amaral FGD. Melatonin as a hormone: new physiological and clinical insights. Endocr Rev. 2018;39:990–1028. 30215696 10.1210/er.2018-00084

[62] Prado NJ Ferder L Manucha W Diez ER. Anti-inflammatory effects of melatonin in obesity and hypertension. Curr Hypertens Rep. 2018;20:45. 29744660 10.1007/s11906-018-0842-6

